# β-Cyclodextrin-Stabilized Silver Nanoparticle Production Combined with Loop-Mediated Isothermal Amplification for the Visual Detection of Contagious Pathogens

**DOI:** 10.3390/mi15030378

**Published:** 2024-03-12

**Authors:** Rajamanickam Sivakumar, Jae Yoon Byun, Nae Yoon Lee

**Affiliations:** Department of BioNano Technology, Gachon University, 1342 Seongnam-daero, Sujeong-gu, Seongnam-si 13120, Gyeonggi-do, Republic of Korea; sivachem1379@gmail.com (R.S.); wodbs0004@gmail.com (J.Y.B.)

**Keywords:** loop-mediated isothermal amplification, β-cyclodextrin, silver nanoparticles, contagious pathogens, visual detection, point-of-care testing

## Abstract

β-cyclodextrin (β-CD) is a water-soluble, non-toxic, biocompatible, and cage compound that contains six, seven, or eight α-(1–4)-attached D-glucopyranose residues. The hydroxyl group in the β-CD is responsible for the reduction of metal ions as well as stabilizing the nanoparticles. In this study, we developed a colorimetric assay for identifying contagious pathogens such as SARS-CoV-2 and *Enterococcus faecium* (*E. faecium*) via in situ development of β-CD-stabilized silver nanoparticles (AgNPs). In the process, the LAMP amplicons produced a complex with silver nitrate (LAMP amplicon–Ag^+^) which was reduced when heated at 65 °C for 5 min in the presence of β-CD and developed a brown color. The limit of detection was determined to be approximately 10^1^ CFU mL^−1^ and 10 fg µL^−1^ for *E. faecium* and SARS-CoV-2, respectively. Significantly, the colorimetric examination of contagious diseases was completed in less than 50 min, including the LAMP assay and detection process. Owing to the high sensitivity and rapid readout mechanism of the β-CD-stabilized AgNP-based colorimetric assay, it is anticipated that the introduced method can be efficiently utilized as a versatile point-of-care testing (POCT) platform for molecular diagnostics in resource-limited areas.

## 1. Introduction

The spread of contagious pathogens has become one of the biggest global concerns threatening human life. For instance, the current coronavirus disease 2019 (COVID-19) developed by severe acute respiratory syndrome coronavirus 2 (SARS-CoV-2) has led to unfavorable circumstances in human society. SARS-CoV-2 has rapidly mutated into several variant strains (Alpha, Beta, Delta, and Omicron) with varying transmissibility, virulence, and vaccination effectiveness. In particular, the Delta strains of SARS-CoV-2 caused a second wave of infection in India, with an estimated 0.3 million new cases per day, and spread quickly around the world [1]. Considering the unidentified weak or symptomless cases, the overall number of COVID-19 cases may be increased. Even though treatments and vaccines are still being developed, diagnosis is essential to the prevention of infections and the control of the pandemic situation. In addition to creating major issues for public health, the COVID-19 outbreak adversely affects the world economy [2].

Another disease that can be acquired in a healthcare setting is nosocomial infection, often known as hospital-acquired infection (HAI). Indeed, two prevalent enterococci species called *Enterococcus faecium* (*E. faecium*) and *Enterococcus faecalis* are the most often identified multidrug-resistant nosocomial pathogens [3]. Significantly, enterococci possess several virulence factors such as cytolysin, hyaluronidase, aggregation influence, gelatinase movement, and enterococcal surface protein (*esp*) [4]. These virulence factors facilitate the longevity of enterococci in HAIs. In particular, the *esp* gene is a key candidate for pathogenicity in clinical isolates of *E. faecium.* Furthermore, it is associated with the formation of biofilm, enhanced virulence, and stability in the urinary system [5]. Significantly, after contracting an *E. faecium* infection, the human body often exhibits symptoms such as gastrointestinal distress, vomiting, and nausea. Severe *E. faecium* contagion develops meningitis, sepsis, and urinary tract infections [6]. Since *E. faecium* is resistant to several first-line antibiotics such as vancomycin and penicillin, treating the infections caused by *E. faecium* is still challenging [7]. Moreover, over the last ten years, the percentage of *E. faecium* infections has risen annually in developed countries too. Therefore, it is imperative to have a rapid, easy-to-use, sensitive, and inexpensive approach to identifying these pathogens in order to prevent epidemics and minimize social and financial damage [8,9].

Numerous diagnostic techniques including culture, microscopy, and immunological assay enable the effective detection of pathogens [10,11]. Among these techniques, the traditional culture technique is considered a gold standard for identifying contagious microorganisms. However, it demands a lengthy time to obtain the results, making it unsuitable to use in point-of-care testing (POCT). Immunoassays are popular and promising analytical methods that could be efficient for the detection of pathogens due to their high sensitivity and easiness. Nevertheless, these assays are expensive and time consuming, and produce false positive results in certain cases [11]. In terms of considering the high specificity and sensitivity, nucleic acid amplification-based molecular diagnostics approaches have been enormously involved in detecting infectious pathogens [12,13,14]. Polymerase chain reaction (PCR) has emerged as an auspicious technique in the field of disease monitoring due to its reliable results with high sensitivity [15]. However, the bulky thermal cycler and fluorescence-based detection render the entire system expensive, which makes the PCR method undesirable for POCT. Owing to the use of constant temperature and rapidity, loop-mediated isothermal amplification (LAMP) has emerged as the ideal substitute for PCR to detect nucleic acid [16]. Various strategies based on electrochemistry, fluorescence, and colorimetry were established for the identification of LAMP amplicons. In recent times, colorimetric detection has drawn significant interest in the diagnostic sector owing to its affordability and ease of use [17]. Furthermore, colorimetry is a unique method since it allows for direct read-out with the naked eye and does not require sophisticated equipment to identify pathogens, making it suitable for POCT [18].

The most popularly used technique for determining nucleic acid based on visual changes is introducing pH-responsive dyes into LAMP [19,20]. Notably, the pH-responsive dye-based detection methods lower the possibility of contamination since the addition of dyes before amplification means that the naked eye can easily perceive the positive and negative targets [21]. However, the failure to produce adequate pH differences in some targets and the necessity of the low-buffered reagents limit their usage in the LAMP process. Recently, noble metal nanoparticles (AuNPs/AgNPs) served as an alternative to pH-responsive dyes and were introduced for LAMP amplicon detection due to their unique surface plasmon resonance property [22,23]. For AuNP/AgNP-based visual amplicon identification, there are two primary methods: (1) the target-specific (with label) method and (2) the target-independent (label-free) method. The former encompasses the functionalization of AuNPs with oligonucleotides, which act as complementary probes for specifically targeting the nucleic acid. Notably, the limit of detection (LOD) has been achieved by up to 10 copies per reaction using the target-specific approach [24]. However, the need for laborious AuNP surface functionalization and lengthy processes, and the high possibility of contamination, demonstrate the disadvantages of this technique. Indeed, the label-free process mainly depends on the stability of the AuNPs/AgNPs, i.e., dispersion or aggregation state. For instance, the red color of carboxyl-modified AuNPs was sustained in the positive LAMP sample. However, it was aggregated in the negative sample due to the absence of magnesium pyrophosphate [25]. Furthermore, the tetrahedron-functionalized AgNP probe was developed, and the color of the probe changed to dark yellow in the presence of HIV-related DNA [26]. Although many benefits exist with AuNP/AgNP-modified probes for LAMP amplicon detection, altering the nanoparticle surface is more expensive and time consuming, which contradicts the idea of POCT. To avoid this, in situ AgNP production could be a feasible method for LAMP amplicon detection.

**Figure 1:**
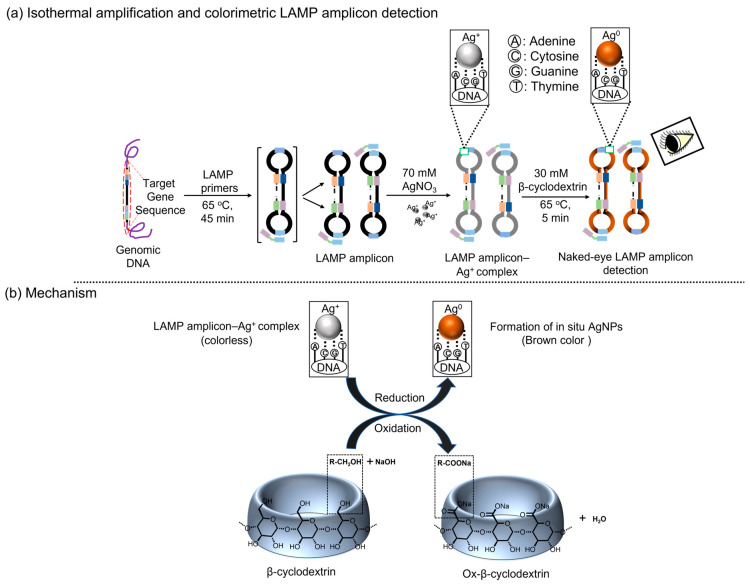
Schematic illustrations showing (**a**) the principles for LAMP and naked-eye DNA detection and (**b**) β-CD-stabilized silver nanoparticle (AgNP)-based LAMP amplicon detection.

In the biological approach, microorganisms have the potential to reduce the silver ions (Ag^+^) to AgNPs [27]. However, when considering low temperature, simplicity, and rapidity, the chemical reduction of Ag^+^ is more beneficial than the biological method [28,29]. Interestingly, the reducing agents can also serve as stabilizing agents, which restricts the aggregation of nanoparticles [30]. Recently, several investigations have shown that noble metal nanoparticles can be stabilized by polymers, surfactants, oligosaccharides, and polysaccharides [29,31]. However, due to the rising demand for the application of environmentally friendly and sustainable techniques, the use of non-toxic and biodegradable materials to stabilize the metal nanoparticles has been widely studied. On this occasion, the cyclodextrins, which belong to the polysaccharides group, can act as reducing as well as stabilizing agents to produce the silver nanoparticles. Moreover, owing to the non-toxic properties of cyclodextrins, they have been widely utilized in various applications such as biocatalysis, cosmetics, drug delivery, packing, and the textile field [32]. Thus, we have introduced β-cyclodextrin (β-CD) to reduce the Ag^+^ to produce brown-colored AgNPs in the presence of LAMP amplicons (Figure 1). Significantly, the colorimetric detection of LAMP amplicons based on the in situ formation of β-CD-stabilized AgNPs has not been examined yet. Hence, in this study, we develop a simple, rapid, and affordable method to visualize LAMP amplicons via AgNP formation. For the proof-of-concept experiment, two major contagious pathogens, *E. faecium* and SARS-CoV-2, were chosen, and the selectivity, as well as the sensitivity, of the introduced method was explored.

### Principle

In DNA, the silver atom typically interacts with the oxygen atom of the deoxyribose unit and phosphodiester bond, whereas Ag^+^ preferentially binds to the nitrogen-contained nucleobases [33,34]. Notably, Cytosine (C) > Guanine (G) > Adenine (A) > Thymine is the order of affinity between Ag^+^ and nucleobases. In particular, the bases of purines (A and G) and pyrimidines (C and T) have served as active sites for incorporating Ag^+^ [35]. The Ag^+^–DNA complex can be reduced to produce AgNPs using β-CD, which is considered an eco-friendly method. Indeed, β-CD eliminates the use of the toxic chemicals typically utilized in traditional methods for nanoparticle synthesis. Moreover, β-CD exhibits greater reactivity in the reduction process due to its internal cavity distance of 7–7.8 Å. Due to the unique structural characteristics of the β-CD molecule, it acts as a remarkable reducing agent during the synthesis of AgNPs [28,36]. Significantly, the primary hydroxyl groups in the β-CD are responsible for denoting the electrons to reduce the Ag^+^ to AgNPs in alkaline conditions (Figure 1b). Furthermore, β-CD can bind to the AgNP surface via chemisorption, which effectively stabilizes the nanoparticles [37].

## 2. Experimental Section

### 2.1. Reagents

β-cyclodextrin (C_15_H_10_O_7_, ≥95%) and silver nitrate (AgNO_3_, ≥99%) were purchased from Sigma-Aldrich (St. Louis, MO, USA). The LAMP kit containing a mixture of dNTP, 10× isothermal amplification buffer, 100 mM magnesium sulfate (MgSO_4_), and *Bst* 2.0 WarmStart DNA polymerase were purchased from New England BioLabs (Ipswich, MA, USA). Sodium hydroxide (NaOH, ≥99%) was purchased from Daejung Chemicals Ltd., Siheung City, Republic of Korea. TE buffer (10 nM Tris-HCl, 0.1 nM EDTA, pH 8.0) was purchased from Thermo Fisher Scientific (Waltham, MA, USA). An FTA card and a purification reagent were purchased from Sigma-Aldrich. The Whatman filter paper was obtained from GE Healthcare Life Sciences, Guangzhou, China. A 100 bp DNA size marker was purchased from Takara (Kusatsu, Japan). Agarose powder for gel electrophoresis was purchased from BioShop (Burlington, ON, Canada). The agar bacteriology and brain heart infusion (BHI) broth were purchased from MBcell (Seoul, Republic of Korea). Ethidium bromide dye was purchased from Dynebio (Seongnam, Republic of Korea). A genomic DNA purification kit was purchased from Promega (Madison, WI, USA). The pDONR207 SARS-CoV-2 E plasmids and *esp* gene of *E. faecium* (ATCC: BAA-2127) were bought from Addgene (Watertown, MA, USA) and ATCC, respectively, and kept at −20 °C. Six sets of LAMP primers, namely, the forward and backward outer primers (F3 and B3), forward and backward inner primers (FIP and BIP), and forward and backward loop primers (LF and LP), were purchased from Cosmo Genetech, Seoul, Republic of Korea.

### 2.2. Instrumentation

An Epoch microplate spectrophotometer (Sulim Science, Seoul, Republic of Korea) was used to record ultraviolet (UV) absorption spectral data. Agarose gel electrophoresis products were photographed in a UV transilluminator purchased from Korea Labtech, Gyeonggi-do, Republic of Korea.

### 2.3. Primer Design

The *esp* gene and pDONR207 SARS-CoV-2 E plasmids were selected as the specific targets for the detection of *E. faecium* and SARS-CoV-2. A set of five primers including two outer primers (F3 and B3), two inner primers (FIP and BIP), and one loop primer (LB) for *E. faecium* and six primers for SARS-CoV-2 were designed using Primer Explorer V5 software (https://primerexplorer.jp/lampv5e/index.html accessed on 16 June 2023). The primer sequences and target genes are shown in Table 1.

### 2.4. Bacterial Cell Culture and DNA Extraction Using FTA Card

In this study, two pathogens, *E. faecium* and SARS-CoV-2, were examined. For the growth of *E. faecium*, liquid culture media and agar plates were employed. The bacteria were grown in 5 mL of BHI broth at 37 °C for 16 h with frequent agitation at 200 rpm. The number of cell colonies generated on the BHI agar plate was counted to determine the bacterial concentrations. The FTA card was employed to extract DNA from the bacterial culture solution. First, the FTA card was loaded with 3 µL of bacterial culture solution and was kept for 30 min at RT. Afterward, the FTA card was treated with 25 µL of purification reagent as well as 25 µL of TE buffer for obtaining pure DNA. Significantly, the concentration of DNA was quantified using NanoDrop 2000 spectrophotometer (Thermo Scientific, Waltham, MA, USA) based on the UV absorbance peak at 260 nm. The UV absorption ratio measured at 260/280 nm was used to calculate the purity of the DNA.

### 2.5. Loop-Mediated Isothermal Amplification Assay

To perform the LAMP assay, the reaction mixture with a total volume of 25 µL containing 10× isothermal amplification buffer (3.5 µL), 1.4 mM dNTP mixture (2.5 µL), 0.8 µM of LB (0.5 µL), 1.6 µM of BIP (0.5 µL) and FIP (0.5 µL), 0.2 µM of F3 (0.5 µL) and B3 (0.5 µL), 6 mM of MgSO_4_ (1.5 µL), 8 U mL^−1^ of *Bst* 2.0 WarmStart polymerase (0.5 μL), target DNA (1 μL), and water (13.5 μL) was prepared in a microtube. The LAMP mixture was then heated for 45 min at 65 °C in order to amplify. Moreover, the LAMP product was visualized using agarose gel electrophoresis stained with ethidium bromide to confirm that the target gene was amplified. Experiments were repeated three times to demonstrate the reproducibility of the method. The LAMP assay for the SARS-CoV-2 E plasmids was as follows: 10× isothermal amplification buffer (3.5 µL), 1.4 mM dNTP mixture (2.5 µL), 0.8 µM of LB (0.5 µL), and LF (0.5 µL), 1.6 µM of BIP (0.5 µL) and FIP (0.5 µL), 0.2 µM of F3 (0.5 µL) and B3 (0.5 µL), 6 mM of MgSO_4_ (1.5 µL), 8 U mL^−1^ of *Bst* 2.0 WarmStart polymerase (0.5 μL), target DNA (1 μL), and water (13 μL).

### 2.6. Colorimetric Detection via β-CD-Stabilized Silver Nanoparticle Formation

The overall colorimetric strategy of the introduced method for detecting LAMP amplicons is depicted in Figure 1a. In a microtube, the LAMP amplicon (5 μL), 70 mM of AgNO_3_ (5 μL), and 30 mM of β-CD in NaOH solution (5 μL) were simultaneously added followed by heating at 65 °C for 5 min for color generation.

### 2.7. Ultraviolet Absorbance Spectral Study of the β-CD-Stabilized Silver Nanoparticles

The UV absorbance spectral measurements of AgNPs were performed in a 96-well plate using an Epoch microplate spectrophotometer. Briefly, 90 µL of water was added to the DNA containing β-CD-stabilized AgNPs, and the resulting solution was thoroughly mixed for 30 s using a pipette. Subsequently, the solution was allowed to incubate for 5 min prior to recording UV–vis absorbance spectra within the wavelength range of 300 to 700 nm.

### 2.8. Agarose Gel Electrophoresis

Agarose gel electrophoresis is a highly effective method for identifying LAMP-amplified products. A gel was prepared using a 1.5% agarose solution. Then, the DNA ladder, negative control, and LAMP amplicons were loaded into the sample wells using loading dye (ethidium bromide). Subsequently, the gel was allowed to run at a specific voltage for 30 min. The gel was then visualized in the UV transilluminator, and the image was recorded.

### 2.9. Sensitivity and Specificity Tests

The sensitivity test was carried out using serially diluted 10-fold *E. faecium* and SARS-CoV-2 E plasmids. LOD was investigated using agarose gel electrophoresis and an AgNP-based visual technique mediated by the use of β-CD as a reducing agent. The target DNA was mixed with or without suitable primers, and the detection process was performed followed by LAMP to verify the specificity of the AgNP-based visual approach.

## 3. Results and Discussion

### 3.1. Optimization of Critical Experimental Conditions

**Figure 2:**
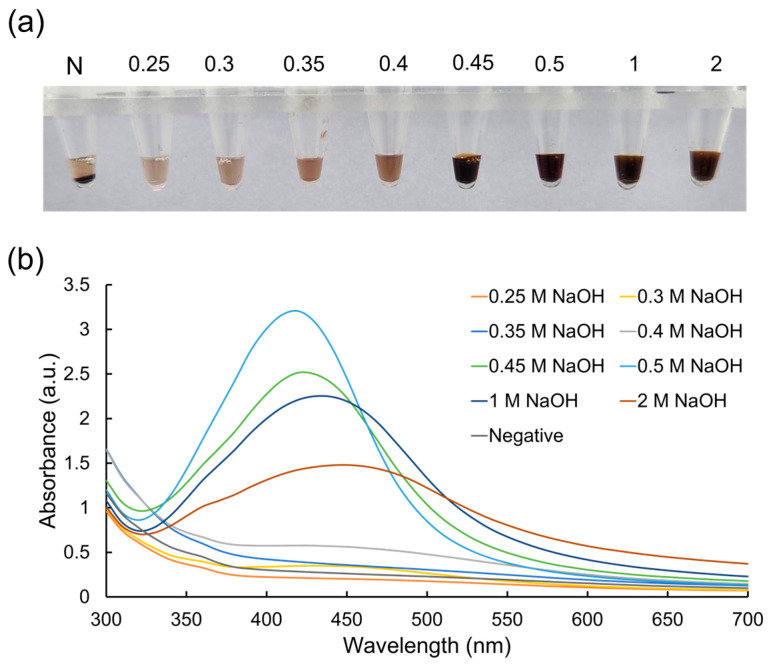
Results showing the optimization of NaOH concentrations for the detection of the LAMP amplicons via (**a**) colorimetry and (**b**) UV–vis spectrophotometry.

Numerous experiments were carried out to optimize the AgNP-based colorimetry process for LAMP amplicon detection by altering reaction parameters such as the concentration of the reactants (AgNO_3_, β-CD, and NaOH) and time. Initially, the concentrations of AgNO_3_ (70 mM) and β-CD (30 mM) were kept constant, whereas different concentrations of NaOH were introduced to initiate the reaction. In this instance, eight distinct NaOH concentrations from 0.25 to 2 M were selected. The UV–vis absorption spectra of the AgNPs produced at various NaOH concentrations are displayed in Figure 2. When the concentration of NaOH was adjusted between 0.25 and 2 M, the absorption intensities of the AgNPs measured at 420 nm varied, which may be due to the size of the produced nanoparticles. Based on the visual inspection and UV–vis absorption spectra, all the negative samples did not develop AgNPs in the ranges of the NaOH concentrations tested from 0.25 to 2 M. Significantly, AgNPs were not produced for NaOH concentrations ranging between 0.25 and 0.4 M in the presence of LAMP amplicons because of inadequate basicity. Notably, when increasing the NaOH concentration from 0.4 to 2 M, the LAMP–Ag^+^ complex was reduced, and well-dispersed AgNPs were produced. However, when the concentrations of NaOH were higher, NaOH reacted with AgNO_3_ and developed a silver hydroxide. Thus, the produced AgNPs at 1 and 2 M NaOH showed lower intensity when compared with 0.5 M of NaOH. These results suggested that 0.5 M NaOH was the ideal concentration, which was used in further experiments.

**Figure 3:**
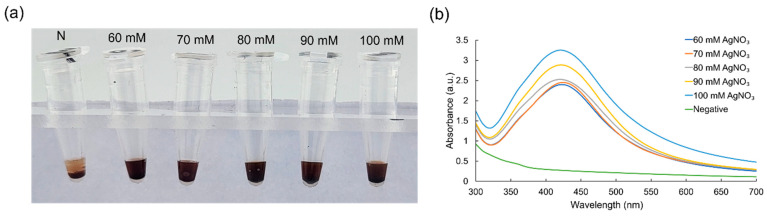
Results showing the optimization of AgNO_3_ concentrations for the detection of the LAMP amplicons via (**a**) colorimetry and (**b**) UV–vis spectrophotometry.

To better understand the impact of the concentration of the metal precursor and reducing agent in the colorimetric LAMP assay, the concentrations of AgNO_3_ were varied while maintaining the concentration of β-CD at 30 mM. The colorimetry image and UV–vis spectra of the AgNPs produced at various AgNO_3_ concentrations are shown in Figure 3a,b. Evidently, the UV absorbance peak at 430 nm was observed in all the concentrations from 60 to 100 mM of AgNO_3_. Significantly, the intensity was directly proportional to the concentrations of AgNO_3_. Since AgNPs were produced in all the concentrations ranging from 60 to 100 mM, any concentrations could be used, and 70 mM AgNO_3_ was selected for further experiments.

### 3.2. Results of the Sensitivity Assay

**Figure 4:**
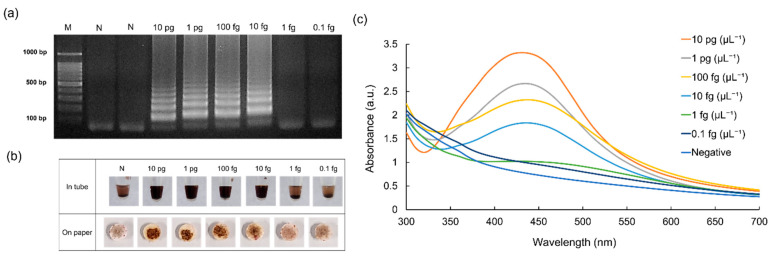
Results showing the sensitivity test for the detection of SARS-CoV-2 E plasmids. Results of (**a**) agarose gel electrophoresis, (**b**) AgNP-based visual detection performed on a microtube and paper, and (**c**) UV–vis spectrophotometry.

The sensitivity of the AgNP-based colorimetry for detecting SARS-CoV-2 E plasmids is shown in Figure 4. The original concentration of SARS-CoV-2 E plasmids (10 pg µL^−1^) was serially diluted 10-fold, down to 0.1 fg µL^−1^. When the agarose gel electrophoresis was performed using the LAMP amplicons, ladder-like bands appeared down to 10 fg µL^−1^, indicating that a low concentration of DNA was successfully amplified (Figure 4a). Notably, the performance of the introduced visual detection strategy was comparable to those obtained using agarose gel electrophoresis (Figure 4b), and the LOD was determined to be 10 fg µL^−1^ for SARS-CoV-2 E plasmids. Significantly, the paper disk was also employed for the sensitivity test, which showed as similar to the microtube results (Figure 4b). A UV–vis spectrophotometer was used to validate the AgNP formation (Figure 4c). It is commonly recognized that the degree of interparticle coupling determines the SPR shift of the metal nanoparticles. Furthermore, the distance between the nearby metal nanoparticles can determine the magnitude of the plasmon shift [38]. Figure 4c shows that the concentration of the DNA has a significant effect on the stability of AgNPs. Specifically, at higher DNA concentrations, AgNPs remain well dispersed owing to the stabilization effect of DNA which is afforded by the negatively charged phosphate groups. Furthermore, when the concentration of DNA was lowered, the absorbance peak of AgNPs at 430 nm gradually moved to the red shift. Hence, it is evident that the absorbance maxima of AgNPs are centered at approximately 470 nm at lower DNA concentrations, indicating the development of aggregated AgNPs. As shown in Figure 4c, the highest concentration of DNA tested in this experiment, 10 pg µL^−1^, attained the maximum absorbance intensity, and there was a strong correlation between the concentration of the DNA and the intensity peak. Moreover, no absorbance peak was developed for 0.1 fg µL^−1^ and the negative sample. Thus, the LOD for SARS-CoV-2 E plasmids was determined to be 10 fg µL^−1^. This analytical result demonstrated the accuracy of the AgNP-based colorimetric findings.

In recent times, versatile techniques such as immunoassays, surface plasmon resonance (SPR)-based methods, and pH-responsive methods have been frequently employed for COVID-19 diagnosis [39,40,41,42,43]. However, the need for the use of costly antigens/antibodies and the functionalization of oligonucleotides make these methods unattractive. In contrast, the color was generated in an in situ manner in the introduced method by simply reducing the DNA–Ag^+^ complex. As a result, AgNPs combined with the LAMP amplicons may provide an alternative to existing colorimetric methods, expanding their potential use in resource-constrained environments. Moreover, the introduced method could be applied in paper-based microfluidics to identify contagious pathogens in an economical manner.

**Figure 5:**
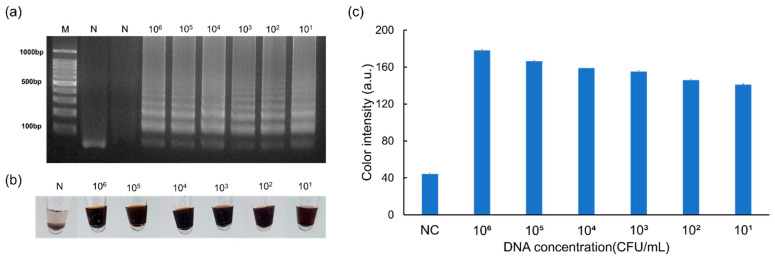
Results of the sensitivity test for the detection of *E. faecium*. Results of (**a**) agarose gel electrophoresis, (**b**) AgNP-based visual detection performed on a microtube, and (**c**) quantification of color intensity.

*E. faecium*, another infectious pathogen, was used to examine the versatile nature of the introduced method. In both colorimetric and agarose-gel-electrophoresis-based detection, the LOD was found to be 10^1^ CFU mL^−1^ (Figure 5a–c). Notably, the detection process was completed within 5 min, which verified the significance of the AgNP-based colorimetric technique mediated by β-CD. Moreover, since the colorimetric substances were more stable and less expensive, the approach presented in this work is highly attractive. Therefore, on-site pathogen detection could be successfully performed using the presented approach. A comparison of the AgNP-based colorimetric detection with other pertinent methodologies for the detection of infectious pathogens is displayed in Table 2.

### 3.3. Results of the Selectivity Assay

**Figure 6:**
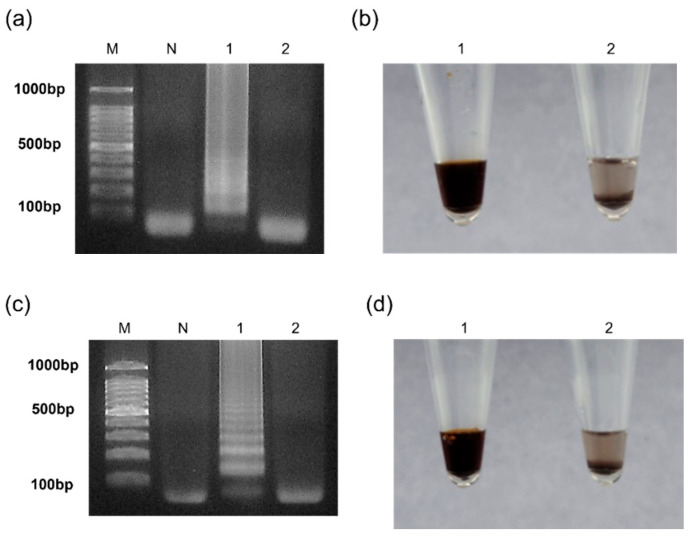
Results of the selectivity test. Results of agarose gel electrophoresis for SARS-CoV-2 E plasmids and *E. faecium* (**a**,**c**) and AgNP-based visual detection for SARS-CoV-2 E plasmids and *E. faecium* (**b**,**d**). (**a**,**b**) 1: SARS-CoV-2 E plasmids with their appropriate primers; 2: SARS-CoV-2 E plasmids with *E. faecium* primers; (**c**,**d**) 1: *E. faecium* DNA with their appropriate primers; 2: *E. faecium* DNA with SARS-CoV-2 E plasmids primers. M: 100 bp DNA ladder; N: Negative.

Since cross-reactivity is one of the main factors used to discriminate false positives, all molecular diagnostic methods must employ primers not displaying cross-reactivity. Significantly, the three sets of primers that were precisely designed to detect the target DNA could be attributed to the excellent selectivity of the LAMP. Two targets were chosen to evaluate the selectivity of the introduced method—SARS-CoV-2 E plasmids and the *esp* gene of *E. faecium*. As shown in Figure 6a,b, the SARS-CoV-2 E plasmids were amplified only when their appropriate primers were presented, and the selectivity towards *E. faecium* was also examined in a similar manner. The obtained results were confirmed by agarose gel electrophoresis as well as the AgNP-based colorimetric technique (Figure 6c,d). To evaluate repeatability, the tests were conducted three times, and consistent outcomes were achieved in each experiment.

## 4. Conclusions

In this work, we developed an in situ AgNP-based strategy mediated by the use of β-CD as a reducing agent for the visual detection of two infectious pathogens, SARS-CoV-2 and *E. faecium.* The introduced method allowed the naked eye to distinguish between positive and negative samples and eliminated the need for the use of expensive equipment to monitor the outcomes. The visual inspection of contagious diseases was accomplished in less than 50 min, including the LAMP assay (45 min) and detection process (5 min), using only an ordinary heat block. The AgNP-based colorimetric technique exhibited remarkable sensitivity to *E. faecium* and SARS-CoV-2, with LODs of 10^1^ CFU mL^−1^ and 10 fg µL^−1^, respectively. Owing to the simplicity, rapidity, and sensitivity, we strongly believe that the visual detection realized via β-CD-stabilized AgNP formation could be an effective alternative to existing colorimetric approaches for on-site identification of infectious pathogens in the POCT platform in resource-limited settings.

## Figures and Tables

**Table 1:** Target genes and primer sequences used in this study.

Target Gene	Primer	Primer Sequences (5′-3′)
*esp* gene (*E. faecium*)	LB	TGATGTTGACACAACAGTTAAGGG
	F3	CCAGAACACTTATGGAACAG
	B3	GTTGGGCTTTGTGACCTG
	FIP	CGTGTCTCCGCTCTCTTCTTTTTATTTGCAAGATATTGATGGTG
	BIP	ATCGGGAAACCTGAATTAGAAGAAGAACTCGTGGATGAATACTTTC
E gene (SARS-CoV-2)	LF	AGGAACACCACGAAGGCC
	LB	ACTGCTGCAACATCGTGAAC
	F3	GAAACCGGCACCCTGATC
	B3	GGAGCTGTTCAGGTTCTTCA
	FIP	TCAGGATAGCCAGGGTCACCAGTGAACTCCGTGCTGCTCT
	BIP	CGCTCTGAGACTGTGCGCTTCGCGGCTGTACACGTAGA

**Table 2:** Comparison of the introduced method with other relevant methodologies for the detection of contagious pathogens.

Target	Sensing Strategies	Sensitivity	Total Assay Time	Ref.
*E. faecalis*	Colorimetry	10 pg	60 min	[44]
*S. Typhimurium*	Colorimetry	50 CFU	80 min	[45]
*S. aureus*	Colorimetry	10^4^ CFU g^−1^	65 min	[46]
*C. jejuni*	Colorimetry	8 CFU mL^−1^	120 min	[47]
SARS-CoV-2	Colorimetry	200 copies mL^−1^	60 min	[48]
*S. iniae*	SPR	10^2^ CFU	120 min	[49]
*S. aureus*	SPR	10 CFU g^−1^	60 min	[17]
*E. coli*	Fluorescence	200 copies	85 min	[50]
*V. parahaemolyticus*	Fluorescence	10^4^ copies μL^−1^	90 min	[51]
SARS-CoV-2	Colorimetry	10 fg µL^−1^	50 min	This work
*E. faecium*	Colorimetry	10^1^ CFU mL^−1^	50 min	This work

## Data Availability

Data are contained within the article.
